# Effect of tranexamic acid administration on intraoperative blood loss during peritonectomy: a single-center retrospective observational study

**DOI:** 10.1186/s40981-023-00631-x

**Published:** 2023-06-22

**Authors:** Daiki Shirasu, Masahiko Tsuchiya, Noriaki Oomae, Wataru Shirasaka, Tatsuhiko Iino, Daisuke Hirano, Makoto Satani

**Affiliations:** 1 Kishiwada Tokushukai Hospital, Emergency and Critical Care Center, 4-27-1 Kamori-Cho, Kishiwada, Osaka 596-0042 Japan; 2 Department of Anesthesiology, Kishiwada Tokushukai Hospital, 4-27-1 Kamori-Cho, Kishiwada, Osaka 596-0042 Japan; 3 Department of Orthopedics, Kishiwada Tokushukai Hospital, 4-27-1 Kamori-Cho, Kishiwada, Osaka 596-0042 Japan

**Keywords:** Anesthesia, Tranexamic acid, Peritoneal pseudomucinoma, Cancerous peritoneal dissemination, Blood transfusion

## Abstract

**Background:**

The efficacy of tranexamic acid in elective major invasive abdominal surgeries has not yet been established. We investigated the effect of tranexamic acid administration on intraoperative blood loss during peritoneal resection of pseudomucinoma and cancerous peritoneal dissemination.

**Methods:**

Patients aged ≥ 20 years old who underwent peritoneal resection for pseudomucinoma or cancerous peritoneal dissemination at the Kishiwada Tokushukai Hospital were included in this single-center retrospective observational study. The tranexamic acid group received 1000 mg of tranexamic acid at the start of the operation, while the control group received the same intraoperative management as the tranexamic acid group, except for the tranexamic acid administration. The primary endpoint was intraoperative blood loss, and a multivariate analysis of the contributing factors was performed.

**Results:**

The median volume of intraoperative blood loss was 1372 [interquartile range, 842 − 1877] mL and 907 [516 − 1537] mL in the control and tranexamic acid groups, respectively (*p* < 0.01). The total volume of blood transfusion during the operation was 2040 [1480 − 2380] mL and 1560 [1000 − 2120] mL in the control and tranexamic acid groups, respectively (*p* = 0.02). Postoperative blood test results revealed D-dimer values of 7.5 [4.1 − 10.7] µg/mL and 1.8 [1.0 − 3.3] µg/mL in the control and tranexamic acid groups, respectively (*p* < 0.01). Multivariate analysis showed that tranexamic acid administration was significantly associated with decreased intraoperative blood loss (*p* = 0.02).

**Conclusion:**

Tranexamic acid administration may be useful in reducing intraoperative blood loss and blood transfusion volume during highly-invasive surgeries such as peritoneal resection of pseudomucinoma and cancerous peritoneal dissemination.

## Background

Tranexamic acid binds to plasminogen and inhibits the binding of plasminogen to fibrin, causing inhibition of fibrinolysis and hemostasis. In orthopedics, cardiac surgery, and perinatology, the use of tranexamic acid has been shown to reduce blood loss and the need for blood transfusion [1–4]. Furthermore, it has been shown to improve the life outcomes of patients with acute trauma, including abdominal surgeries [5].

The effect of tranexamic acid in elective abdominal surgeries has not yet been established. Previous studies have reported no difference in blood loss intra- and postoperatively [6, 7], while others have reported a decrease in blood loss [6, 8]. Recently, tranexamic acid has been reported to reduce severe blood loss during non-cardiac and non-cerebral surgeries [9]. However, its effect on abdominal surgeries with massive bleeding was not established. Peritonectomy for pseudomucinoma or cancerous peritoneal dissemination is relatively uncommon in Japan; however, it is a good option that is widely performed in Europe and China [10]. A certain amount of bleeding and plasma loss is expected during peritoneal resection surgery based on our previous experience with the surgery. To our knowledge, blood loss during peritonectomy with tranexamic acid has not been reported. This study aimed to investigate the effect of tranexamic acid on bleeding during this highly invasive surgery and the changes in coagulation and fibrinolysis activities after the surgery.

## Methods

This was a single-center, retrospective, observational study. The study protocol was approved by the Ethics Committee of the Kishiwada Tokushukai Hospital (Approval number 22–05, Approval date March 14, 2022). The requirement of obtaining written informed consent from each individual was omitted by disclosing the information on the hospital’s website in accordance with the Japanese guidelines. This study was conducted in accordance with the principles of the Declaration of Helsinki. We followed the applicable EQUATOR Network (http://www.equator-network.org/) guidelines, specifically the STROBE Guideline, during this research project.

We included patients aged ≥ 20 years who underwent peritoneal resection for pseudomucinoma or cancerous peritoneal dissemination at the Kishiwada Tokushukai Hospital. The study period was from December 1, 2018, to December 31, 2021. We excluded patients with coagulation abnormalities before the surgery (prothrombin activity < 75%, activated partial thromboplastin time ratio > 1.6, or platelet count < 15.8 × 10^4^/µL) and patients weighing < 40 kg. Patients who received albumin preparations intraoperatively were also excluded. Each patient underwent ultrasound examination to confirm the absence of venous thrombi in the lower extremities before the surgery.

The tranexamic acid group received 1000 mg of tranexamic acid at the start of the surgery. Intraoperative management in the control group was the same as in the tranexamic acid group, except that tranexamic acid was not used. The decision to administer tranexamic acid was made by the anesthesiologist, and the surgeon was not notified. Blood transfusions were performed according to the criteria below. Red cell concentrates were administered when blood loss exceeded 300 mL and further bleeding was expected. Fresh frozen plasma was administered in doses of 4–6 units (equivalent to 480 − 720 mL) early in the operation, regardless of bleeding. Platelet concentrates were administered when the platelet count fell below 70,000/µL as per blood test results.

The primary endpoint was intraoperative blood loss. The secondary endpoints included the volume of blood transfused intra- and postoperatively; blood test results of hemoglobin, coagulation, and fibrinolysis immediately after the surgery and on postoperative day 1; operation time; length of hospital stay; and 28-day mortality. The transfusion rate was defined as the ratio of the number of patients who received any unit of blood components.

### Statistical analysis

Fisher’s exact test was used to compare nominal variables in the two independent groups. The Mann − Whitney U test was used to compare ordinal and non-normally distributed continuous variables in the two independent groups, and the t-test was used to compare normally distributed continuous variables in the two independent groups. Data are expressed as proportions, medians and interquartile range, and mean and standard deviation, as appropriate. A two-sided *p* < 0.05 was considered statistically significant. Missing values were not filled in, and statistical processing was performed accordingly.

Furthermore, we performed multiple regression analysis to determine the association between the amount of intraoperative blood loss and tranexamic acid administration. In the analysis, we used log-transformed intraoperative blood loss as the objective variable so that it would be normally distributed. Tranexamic acid administration, sex, age, operative time, and whether hyperthermia chemotherapy was administered were considered explanatory variables.

All statistical analyses were performed using EZR (Saitama Medical Center, Jichi Medical University, Saitama, Japan), a graphical user interface for R (The R Foundation for Statistical Computing, Vienna, Austria) [11].

## Results

The total number of patients was 134 (60 and 74 in the control and tranexamic acid groups, respectively). Their background characteristics are presented in Table 1. There was no difference in the patient background between the two groups, except for sex (*p* = 0.01). The primary diseases included 44 cases of appendiceal cancer (32.8%), 23 cases of colorectal cancer (17.2%), 20 cases of gastric cancer (14.9%), 18 cases of ovarian cancer (13.4%), and others.

**Table 1:** Patient background

		Control group (*N* = 60)	Tranexamic acid group (*N* = 74)	*P*-value
Sex	Male	29 (48.3)	19 (25.7)	0.01^a^
	Female	31 (51.7)	55 (74.3)	
Age, years		54.8 ± 13.2	55.3 ± 11.2	0.81
Height, cm		162 ± 8	161 ± 11	0.35
Weight, kg		57.6 ± 12.7	55.9 ± 10.8	0.39
Preoperative blood test	Platelet, × 10^4^/µL	25.3 ± 6.1	26.8 ± 6.9	0.19
	APTT ratio	1.02 ± 0.10	1.02 ± 0.10	0.72
	PT, %	104 ± 16	107 ± 13	0.33
	Hemoglobin, g/dL	11.7 ± 1.8	11.8 ± 1.5	0.79
Hyperthermia chemotherapy during the operation		40 (66.7)	40 (54.1)	0.15

Data are expressed as mean ± standard deviation or number (%). ^a^indicates significant difference

*APTT* Activated partial thromboplastin time, *PT *Prothrombin time

Tables 2 and 3 present the results for the primary and secondary endpoints. Intraoperative blood loss was significantly lesser in the tranexamic acid group than that in the control group (*p* < 0.01). The total volume of blood products (including red cell concentrate, fresh frozen plasma, and platelet concentrate) transfused intraoperatively was significantly lesser in the tranexamic acid group (*p* = 0.02). In each blood product comparison, the volume of fresh frozen plasma transfused during the operation was significantly lesser in the tranexamic acid group (*p* < 0.01), while there were no differences in the volume of red cell concentrate between the two groups (*p* = 0.07). Postoperatively, there was no difference in the volume of blood transfusions between the two groups. In addition, there was no difference in the rate of blood transfusions between the two groups, either intra- or postoperatively. The D-dimer level was significantly lower in the tranexamic acid group than that in the control group: 1.8 [1.0–3.3] vs 7.5 [4.1–10.7] µg/mL, *p* < 0.01 and 7.4 [3.8–12.3] vs 18.1 [9.0–24.9] µg/mL, *p* < 0.01 on postoperative days 0 and 1, respectively. The hemoglobin levels were not different in the two groups immediately after the surgery (*p* = 0.22) and on postoperative day 1 (*p* = 0.21). There were no differences in operation time (*p* = 0.05), length of hospital stays (*p* = 0.46), or 28-day mortality (*p* = 0.45) between the two groups.

**Table 2:** Intraoperative blood loss, coagulation study, operation time, and mortality

		Control group (*N* = 60)	Tranexamic acid group (*N* = 74)	*P*-value
Intraoperative blood loss, mL		1372 [842 − 1877]	907 [516 − 1537]	< 0.01^a^
Blood test just after operation	Platelet, × 10^4^/µL	17.1 ± 5.0	18.4 ± 5.7	0.20
	Hemoglobin, g/dL	12.2 ± 1.9	11.8 ± 1.8	0.22
	APTT	1.08 ± 0.49	1.04 ± 0.19	0.54
	PT, %	92 ± 12	93 ± 10	0.82
	Fibrinogen, mg/dL	235 ± 51	234 ± 41	0.88
	D-dimer, µg/mL	7.5 [4.1 − 10.7]	1.8 [1.0 − 3.3]	< 0.01^a^
Blood test on postoperative day 1	Platelet, × 10^4^/µL	15.6 ± 4.8	16.8 ± 5.5	0.18
	Hemoglobin, g/dL	12.1 ± 2.0	11.8 ± 1.6	0.21
	APTT	1.17 ± 0.22	1.16 ± 0.14	0.71
	PT, %	83 ± 11	86 ± 11	0.11
	Fibrinogen, mg/dL	302 ± 55	310 ± 55	0.39
	D-dimer, µg/mL	18.1 [9.0 − 24.9]	7.4 [3.8 − 12.3]	< 0.01^a^
Operation time, minute		240 ± 72	215 ± 77	0.05
Length of hospital stay, day		25 [16 − 35]	20 [16 − 33]	0.46
28-day mortality		1 (1.7)	0 (0)	0.45

Data are expressed as median [interquartile range], mean ± standard deviation, or number (%), without specific indication. ^a^indicates significant difference

*APTT *Activated partial thromboplastin time, *PT *Prothrombin time, *RCC *Red cell concentrates, *FFP *Fresh frozen plasma

**Table 3:** Blood transfusion rate and volume

		The control group (*N* = 60)	The tranexamic acid group (*N* = 74)	*P*-value
Intraoperative				
Blood transfusion rate	RCC	50 (83.3)	56 (75.7)	0.39
	FFP	60 (100)	68 (91.9)	0.06
	PC	0 (0)	2 (2.7)	0.50
Blood transfusion volume^a^	RCC, mL	840 [490 − 1120]	560 [280 − 1120]	0.07
	FFP, mL	1200 [960 − 1200]	1200 [720 − 1200]	< 0.01*
	PC, mL	0 [0 − 0]	0 [0 − 0]	0.20
	Total volume of blood products, mL	2040 [1480 − 2380]	1560 [1000 − 2120]	0.02*
Postoperative				
Blood transfusion rate	RCC	11 (18.3)	17 (23.0)	0.53
	FFP	24 (40.0)	32 (43.2)	0.73
	PC	2 (3.3)	2 (2.7)	1.00
Blood transfusion volume	RCC, mL	0 [0 − 0]	0 [0 − 0]	0.46
	FFP, mL	0 [0 − 240]	0 [0 − 480]	0.35
	PC, mL	0 [0 − 0]	0 [0 − 0]	0.81
	Total volume of blood products, mL	0 [0 − 480]	0 [0 − 550]	0.27

Data are expressed as median [interquartile range] or number (%), without specific indication. * ^a^indicates significant difference

*RCC* Red cell concentrates, *FFP* Fresh frozen plasma, *PC* Platelet concentrates

^a^One unit of PC consists of platelets isolated from 200 mL of blood. It is equivalent to approximately 20 mL and contains > 0.2 × 10^11^ platelets

Table 4 presents the results of the multiple regression analysis performed to examine the relationship between the amount of intraoperative blood loss and the use of tranexamic acid. The administration of tranexamic acid was independently associated with decreased intraoperative blood loss (95% confidence intervals [CI] -0.18 − -0.01, *p* = 0.02). In addition, the analysis demonstrated that operation time and the use of hyperthermia chemotherapy were associated with the amount of intraoperative blood loss (95%CI 0.003 − 0.004, *p* < 0.01 and 95% CI -0.31 − -0.13, *p* < 0.01).

**Table 4:** Results of multiple regression analysis for intraoperative bleeding

Intercept	Regression coefficient	95% CI	*P*-value
Age	-0.0007	-0.004 − 0.003	0.64
Operation time	0.003	0.003 − 0.004	< 0.01^a^
Male	0.01	-0.07 − 0.10	0.69
Tranexamic acid administration	-0.09	-0.18 − -0.01	0.02^a^
Hyperthermia chemotherapy	-0.22	-0.31 − -0.13	< 0.01^a^

*95% CI* 95% confidence intervals

^a^Indicates statistically significant

## Discussion

In this study, we found that the administration of tranexamic acid reduced intra-operative blood loss and blood transfusion volume in peritoneal resection for pseudomucinoma and cancerous peritoneal dissemination. Tranexamic acid forms a complex with plasminogen and exerts its antifibrinolytic effect by preventing the adsorption of plasminogen to fibrin. This mechanism suggests that the hemostatic effect of tranexamic acid is mainly due to venous bleeding or bleeding sites where a hematoma is already forming. In non-cardiac and non-cerebral surgeries, the effect of tranexamic acid on bleeding was observed in life-threatening hemorrhages and bleeding in vital organs [9]. However, in elective abdominal surgeries, the effects remain controversial. Many of the studies were conducted in patients with relatively low intraoperative blood loss [6–8]. Thus, we investigated the effect of tranexamic acid on peritonectomy with massive bleeding and found that its administration greatly reduced intraoperative blood loss. Furthermore, the total amount of blood transfused during the operation was lower in the tranexamic acid group, although there was no difference between the two groups in the rate of blood transfusion. Lower D-dimer levels in the tranexamic acid group after the operation also indicates the inhibitory effects of tranexamic acid on fibrinolysis, as previously reported [12].

Multiple regression analysis demonstrated that longer operation time increased the risk of intraoperative bleeding. This may indicate that the alleviating effect of tranexamic acid on surgical bleeding is rather smaller than the magnitude of surgical invasion of peritonectomy. The analysis also demonstrated that hyperthermia chemotherapy reduced intraoperative blood loss. This characteristic therapy irrigated several liters of saline solution into the abdominal cavity for tens of minutes, which exerted significant water pressure against the abdominal wall. The compression effect might be related to the reduction of bleeding in the abdominal cavity.

The first limitation of this study is that we transfused 4–6 units (equivalent to 480 − 720 mL) of fresh frozen plasma from the start of the surgery for peritonectomy. In the past, we did not use fresh frozen plasma intraoperatively; however, postoperative blood tests showed dilutional coagulopathy and decreased fibrinogen levels. Therefore, our current policy is to transfuse fresh frozen plasma during the early stage of surgery. Nevertheless, our study showed a significant difference in the amount of fresh frozen plasma in the control and tranexamic acid groups intraoperatively. If transfusion therapy were performed in accordance with the guidelines for blood component transfusions by the Japanese Ministry of Health, Labor and Welfare [13], the difference in the amount of intraoperative fresh frozen plasma used between the control and tranexamic acid groups would be much larger. Second, tranexamic acid was administered based on the decision of the anesthesiologist. Compared with randomized controlled trials, the possibility of patient bias in the number of eligible patients cannot be ruled out. Third, the present study found no association between tranexamic acid administration and improved 28-day mortality. Massive bleeding and corresponding blood transfusion could affect the survival rate of surgical patients in the long run [14], which will be our future research target. Fourth, this study did not examine the adverse effects of tranexamic acid administration. Previous studies have reported a dose-dependent increase in the risk of thromboembolism and seizures [15, 16]. However, the usual therapeutic dose of tranexamic acid is not associated with these risks [16, 17]. Therefore, in the current study, the benefit of the administration of 1000 mg of tranexamic acid in reducing intraoperative blood loss outweighed the risk of side effects.

## Conclusion

In peritoneal resection for pseudomucinoma and cancerous peritoneal dissemination, a representative of highly-invasive surgeries, tranexamic acid administered at the start of the surgery is effective for reducing intraoperative blood loss and blood transfusion volume.

## Data Availability

The datasets used and/or analyzed during the current study are available from the corresponding author upon reasonable request.
